# Open Science and Software Assistance: Commentary on “Artificial Intelligence Can Generate Fraudulent but Authentic-Looking Scientific Medical Articles: Pandora’s Box Has Been Opened”

**DOI:** 10.2196/49323

**Published:** 2023-05-31

**Authors:** Pedro L Ballester

**Affiliations:** 1 Neuroscience Graduate Program McMaster University Hamilton, ON Canada

**Keywords:** artificial intelligence, AI, ChatGPT, open science, reproducibility, software assistance

## Abstract

Májovský and colleagues have investigated the important issue of ChatGPT being used for the complete generation of scientific works, including fake data and tables. The issues behind why ChatGPT poses a significant concern to research reach far beyond the model itself. Once again, the lack of reproducibility and visibility of scientific works creates an environment where fraudulent or inaccurate work can thrive. What are some of the ways in which we can handle this new situation?

## Introduction

The potential of ChatGPT to revolutionize science is paramount. That is, for better or for worse. In the recent paper “Artificial Intelligence Can Generate Fraudulent but Authentic-Looking Scientific Medical Articles: Pandora’s Box Has Been Opened,” Májovský and colleagues [1] decided to investigate what happens when ChatGPT is used to generate a complete paper, from the title to the references. This is a commendable and timely work.

Unsurprisingly, given the quality of its language generation, ChatGPT was able to write a convincing paper that for most researchers, apart from experts in the field, is indistinguishable from a human-made research paper. The quality of the work, alongside the generative model’s ability to fabricate data that align with and “confirm” its hypotheses, should sound the alarm to research institutions and journals. As the authors aptly described it, Pandora’s box has been opened. So what can, or should, be done about it?

## How Much Is Too Much?

In the conclusion of the paper, the authors briefly point out some of the pros and cons of this technology. Beyond the con of creating completely fabricated articles alongside fabricated data, the authors mention ChatGPT’s potential for improved editing and research. A natural parallel can thus be drawn between ChatGPT and other less sophisticated language tools, such as Grammarly, Gmail suggestions, a thesaurus, or even Google searching, which could substantially improve productivity and writing skills. Given its proportions, ChatGPT’s challenges are not unlike those faced in the past. Thus, the question really is: how much help from technology is too much help?

When writing a manuscript, the use of technology is ubiquitous. When a typo is present, it is automatically highlighted; Googling aspects of the work is never second-guessed; and changes to some awkwardly written sentences are suggested by Grammarly. The line today between what is acceptable and unacceptable help from technology is most commonly drawn at blatant plagiarism. ChatGPT has now created more of a gray area than ever before. As mentioned by the authors, technologies are in place to detect text that was written by ChatGPT. How do these new technologies fit into the current way in which we detect plagiarism, if at all? If an introduction is written by ChatGPT and edited by the author, how much editing is necessary before the passage is considered to no longer be created by ChatGPT? Moreover, why should a text created by ChatGPT be seen as less than when written by humans, when the authors have vetted and agreed with what was written by the model? In fact, this could dramatically speed up science, removing most of the repetitive nature of scientific writing. Additionally, more help from language models when writing manuscripts can also break down barriers that are faced by nonnative speakers. The opportunities provided by ChatGPT to promote equity have also been highlighted by researchers who have shown how ChatGPT can outperform median scores in the MCAT (Medical College Admission Test) [2]. Therefore, language models have too many benefits to be completely removed from scientific development. We should instead strive to coexist, language models and humans, each contributing to what they do best.

A similar philosophy can be seen in the programming world. Programmers are now using ChatGPT to speed up software development. As long as the generated code is double-checked, most programmers have no problem using its generated code. In fairness, the culture of sharing, especially largely repeatable code, has been a big part of the coding culture, with websites such as StackOverflow specializing in it. Likely due to a preference for standardization over personal style, programming is seen as something where copying is mostly accepted, as long as credit is given to the original authors. The difference between scientific writing and programming is large, and that cannot be ignored, but surely there are lessons to be learned from one another.

This is just the start of the influence of these models in our daily lives. These models are quickly improving, with the addition of better prompt engineering and model self-reflection [3,4]. In fact, prompt engineering is growing, and researchers are now dedicated to finding the best ways to tell ChatGPT how to conduct tasks, improving its ability [5]. Soon enough, we will identify prompts that produce much better papers than what is currently being generated by the models, even without significant improvements to the underlying technology. This is particularly relevant when acknowledging that current prompts can already lead to abstracts that fool scientists [6].

## The Call for Open Science

We need more than discussions about ChatGPT in isolation to understand the change in philosophy that needs to happen in research, particularly in the medical sciences. The generation of fake data is of particular concern since reproducibility has never been prioritized. Code sharing is very much optional in most publication venues, and data sharing agreements for reproducing results are as complicated as they have always been. ChatGPT is not the creator of these issues; it instead enables this problem to exist at a much larger scale. Similarly, poor training among journal reviewers in identifying statistical problems and detecting fraudulent work is probably one of the reasons that fake articles from ChatGPT would have so much room to thrive.

Májovský and colleagues [1] have correctly pointed out that there is a need to combat the misuse of artificial intelligence (AI) in scientific research. Personally, I do not believe there is a way to even start this battle until we properly address the issue of poor reproducibility and visibility of research. For now, we should at least start by declaring the extent to which AI has assisted in the writing and analysis of a paper, much like we do for other aspects of the work in the Methods section. That way, readers can make an informed judgment of the work. That being said, it is hard to think of solutions for all the ethical challenges that we will face. Much like Sam Altman, the current CEO of OpenAI, who has famously told investors that asking questions to ChatGPT would help ChatGPT become profitable as a product, we too should use ChatGPT to help us address these difficult questions ahead.

## Abbreviations

AI: artificial intelligence
MCAT: Medical College Admission Test
